# Reduced *PLCG1* expression is associated with inferior survival for myelodysplastic syndromes

**DOI:** 10.1002/cam4.2717

**Published:** 2019-11-21

**Authors:** Masayuki Shiseki, Mayuko Ishii, Mari Miyazaki, Satoko Osanai, Yan‐Hua Wang, Kentaro Yoshinaga, Naoki Mori, Junji Tanaka

**Affiliations:** ^1^ Department of Hematology Tokyo Women's Medical University Tokyo Japan

**Keywords:** common deleted region, del(20q), haploinsufficiency, MDS, PLCG1

## Abstract

The *PLCG1* gene, which encodes the phospholipase C γ1 isoform, is located within the commonly deleted region of the long arm of chromosome 20 (del(20q)) observed in myelodysplastic syndromes (MDS). Phospholipase C is involved in diverse physiological and pathological cellular processes through inositide signaling. We hypothesized that reduced PLCG1 expression because of haploinsufficiency by del(20q) plays a role in the molecular pathogenesis of MDS. Therefore, we analyzed PLCG1 expression in bone marrow mononuclear cells at diagnosis in 116 MDS patients with or without del(20q) by quantitative RT‐PCR to evaluate its clinical significance. The expression level of PLCG1 was significantly lower not only in MDS patients with del(20q) but also in those without del(20q) compared to that of the controls, which suggests that reduced PLCG1 expression is a common molecular event in MDS. Patients in the lowest quartile (Q4) group for PLCG1 expression had lower overall survival (OS) compared to that of other patients (Q1‐Q3) (log‐rank test, *P* = .0004) with estimated median OS times of 22 in the Q4 group and 106 months in the Q1‐3 group. Univariate and multivariate analysis indicated reduced PLCG1 expression (Q4) was associated with lower OS (hazard ratio 2.58, 95% CI 1.35‐4.84, *P* = .0049), which suggests that reduced PLCG1 expression is an independent prognostic factor for OS. In addition, patients were well‐stratified for OS by combining PLCG1 expression level (Q4 vs Q1‐3) and bone marrow blast percentage (5% or more vs less than 5%). Thus, the level of PLCG1 expression at time of diagnosis is a prognostic biomarker for MDS.

## Graphical abstract

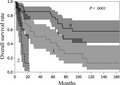

## 1 INTRODUCTION

Myelodysplastic syndromes (MDS) are clonal hematopoietic stem cell disorders characterized by ineffective hematopoiesis and an increased risk of leukemic transformation. Therapeutic options for MDS are limited, and no curative therapy except for allogeneic stem cell transplantation has been established. It is important to elucidate the molecular pathological mechanisms of MDS to develop curative therapies. In approximately half of MDS cases, chromosome abnormalities are found, and monosomy and chromosome deletions are frequently observed. These deleted chromosomal regions contain tumor suppressor genes that are involved in the development and progression of MDS. Decreased expression of these candidate tumor suppressor genes by haploinsufficiency because of chromosome loss may play a role in MDS molecular pathogenesis. Deletion of the long arm of chromosome 20, del(20q), is observed in approximately 5%‐10% of MDS cases.^1^, ^2^ Several research groups, including ours, have examined the common deleted region (CDR) of del(20q) by different molecular biological approaches.^3^, ^4^, ^5^, ^6^, ^7^ We hypothesized that genes located within the CDR are involved in the pathogenesis of MDS because of haploinsufficiency.

The *PLCG1* gene is located within the CDR of del(20q), which were determined by array comparative genomic hybridization.^6^ The *PLCG1* gene encodes phospholipase C γ1, which is involved in diverse physiological and pathological cellular processes by catalyzing the hydrolysis of phosphatidylinositol 4,5‐bisphosphate to generate the second messenger molecules, inositol 1,4,5‐trisphosphate and diacylglycerol.^8^, ^9^, ^10^ Plcg1‐deficient mice do not undergo erythropoiesis and die at an early embryonic stage.^11^ In zebrafish, phospholipase C γ1 is required for granulocyte maturation.^12^ In addition, the nuclear inositide signaling pathway mediated by phospholipase C in MDS is important.^13^ Monoallelic loss of the *PLCB1* gene, which encodes the phospholipase C β1 isoform, another phospholipase C isoform, is often observed in MDS patients and is associated with disease progression.^14^ Decreased PLCB1 expression because of monoallelic loss may cause dysregulation of inositide signaling in MDS. Therefore, we analyzed PLCG1 expression in bone marrow mononuclear cells at diagnosis in MDS patients with or without del(20q) and investigated its clinical significance.

## 2 MATERIALS AND METHODS

### 2.1 Patients and samples

Bone marrow samples taken at the time of MDS diagnosis were used for analysis. Bone marrow samples from 16 patients who were diagnosed as early‐stage malignant lymphoma without evidence of bone marrow involvement were used as controls. In addition to microscopic examination, lymphoma cell involvement in bone marrow was screened by a flow cytometric analysis and a karyotyping in control subjects. Written informed consent was obtained from all patients and control subjects. Mononuclear cells were separated from bone marrow samples and stored in liquid nitrogen until analysis. Data including patients’ demographic, disease status, medical history, clinical and laboratory findings, and outcomes were collected from medical records and a laboratory database.

### 2.2 Quantitative RT‐PCR for PLCG1 expression analysis

To analyze PLCG1 expression, quantitative RT‐PCR was performed. Total RNA was extracted from mononuclear cells and subjected to cDNA synthesis. Real‐time RT‐PCR was performed to quantify the expression level of PLCG1 by the TaqMan probe method (Applied Biosystems) using cDNA as template with co‐amplification of an endogenous control gene human GAPDH (Applied Biosystems). Expression levels of PLCG1 were obtained using the standard curve method in each experiment after normalization by the *GAPDH* gene for each sample in duplicate wells. The expression level of each sample was indicated as a relative ratio to that of K562 cells to standardize different series of experiments.

### 2.3 Statistical analyses

A nonparametric Mann‐Whitney‐Wilcoxon test was used to compare PLCG1 expression levels among groups. The Kaplan‐Meier model was used to analyze the impact of PLCG1 expression on overall survival (OS); a log‐rank test was used for statistical analysis. The Cox proportional hazards model was used to evaluate clinical and biological factors associated with OS. Statistical analyses were performed using JMP Pro version 11.2 (SAS Institute Inc). A significant result was considered as a *P* < .05.

### 2.4 Ethics and study management

The study was conducted in accordance with the Declaration of Helsinki and reviewed by an institutional ethics committee (No. 3426).

## 3 RESULTS

### 3.1 Patients’ characteristics

A total of 116 MDS patients consisting of 69 males and 47 females with a median age of 70 years (range, 20‐91 years) with (n = 23) or without (n = 93) del(20q) were analyzed. Baseline patient characteristics are summarized in Table 1. Patients were classified as 5q‐ (n = 2), RCUD (n = 17), RCMD (n = 54), RARS (n = 10), RAEB‐1 (n = 12), or RAEB‐2 (n = 14) according to the WHO 2008 classification or RAEB‐T (n = 7) according to the FAB classification. Patients were categorized into four international prognostic scoring system (IPSS) risk groups: low risk (n = 31), intermediate‐1 risk (n = 48), intermediate‐2 risk (n = 21), and high risk (n = 13); IPSS risk was unknown in three patients.

**Table 1:** Characteristics of 116 patients

	Del(20q)	Others	Total
	(n = 23)	(n = 93)	(n = 116)
Gender (female/male)	10/13	37/56	69/47
Age (median)	72.5	66	70
(range)	45‐84	20‐91	20‐91
MDS subtypes^a^			
5q‐	0	2	2
RCUD	6	11	17
RARS	2	8	10
RCMD	8	46	54
RAEB‐1	2	10	12
RAEB‐2	3	11	14
RAEB‐T	2	5	7
IPSS			
Low	8	23	31
Intermediate‐1	8	40	48
Intermediate‐2	4	17	21
High	3	10	13
Missing	0	3	3
IPSS‐R			
Very low	4	12	16
Low	6	23	29
Intermediate	4	28	32
High	4	10	14
Very high	5	17	22
Missing	0	3	3
Karyotypes^b^			
Good risk	17	62	79
Intermediate risk	3	8	11
Poor risk	3	21	24
Not determined	0	2	2
Bone marrow blast (median, range) (%)	3.0 (0.3‐25.6)	3.3 (0.6‐27.0)	3.3 (0.3‐27.0)
Neutrophils (median, range) (/μL)	1937 (416‐7045)	1606 (117‐6504)	1705 (117‐7045)
Hemoglobin (median, range) (g/dL)	10.3 (6.9‐14.0)	9.6 (4.3‐13.7)	9.8 (4.3‐14.0)
Platelet count (median, range) (/μL)	8.5 (1.2‐24.7)	10.3 (0.5‐58.2)	9.9 (0.5‐58.2)

Note

The poor risk category included complex abnormalities (three or more abnormalities) and chromosome 7 abnormalities. All other chromosome abnormalities were included in the intermediate risk category.

Abbreviations: IPSS, international prognostic scoring system; IPSS‐R, revised international prognostic scoring system.

a Patients were categorized into subgroups according to the WHO classification in 2008, except for 4 patients (RAEB‐T).

b The good risk category included normal, ‐Y, del(5q), and del(20q).

### 3.2 Significant reduction of PLCG1 expression in bone marrow mononuclear cells of MDS patients

**Figure 1:**
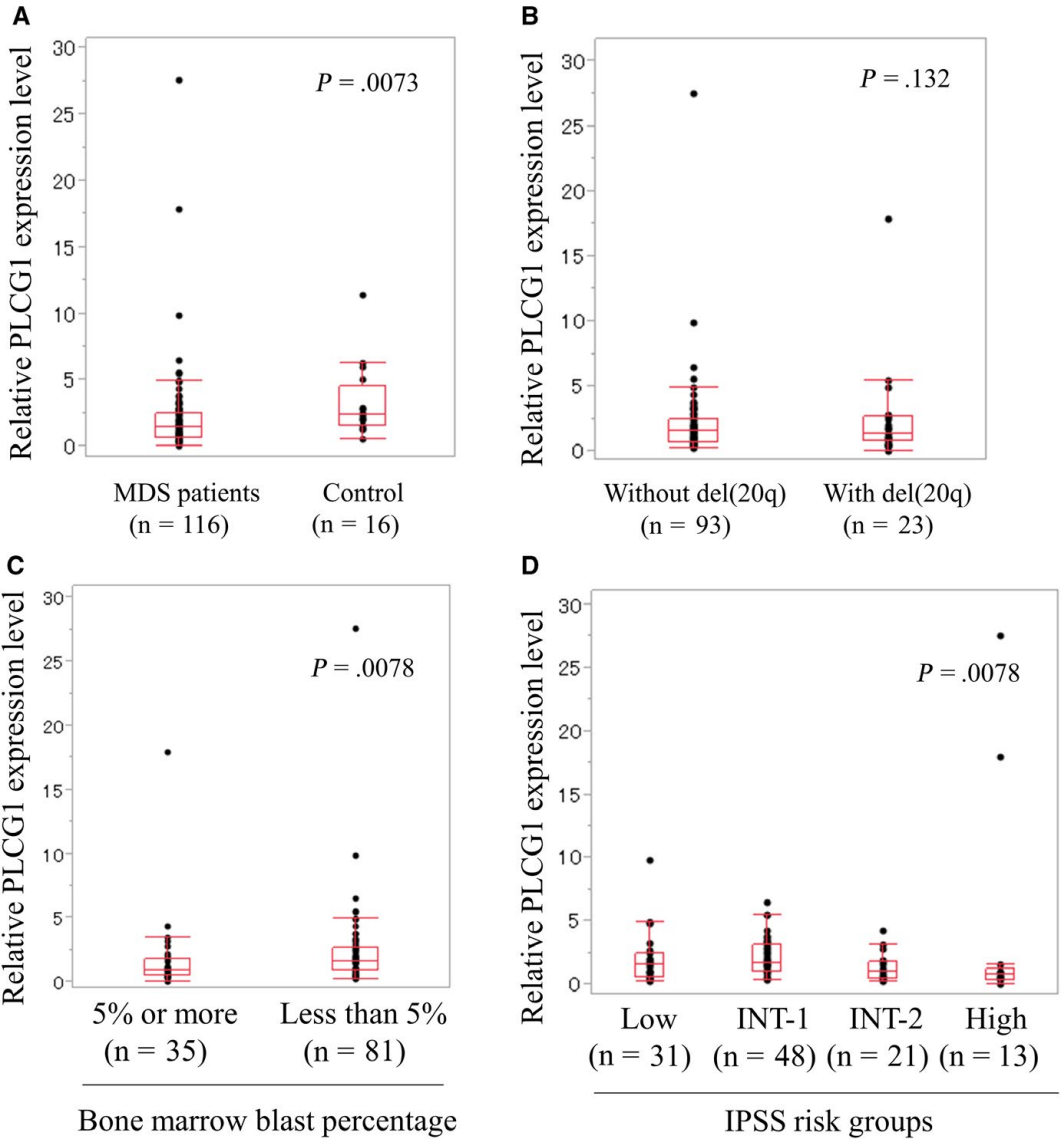
Comparison of relative PLCG1 expression levels between (A) control subjects and myelodysplastic syndromes (MDS) patients, (B) with or without del(20q), (C) among MDS subtypes with high (5% or more) and low (less than 5%) bone marrow blast percentage, and (D) among the four IPSS risk groups

First, we compared PLCG1 expression between MDS patients and control subjects. Relative PLCG1 expression was significantly reduced in 116 MDS patients compared to that of 16 control subjects (*P* = .0073; Figure 1A). The median values of relative PLCG1 expression levels were 1.46 in 116 MDS patients, and 2.38 in control subjects. There was no significant difference in PLCG1 expression between MDS patients with del(20q) (n = 23) and those without del(20q) (n = 93) (*P* = .132; Figure 1B). The median values of relative PLCG1 expression levels were 1.36 in MDS patients with del(20q) and 1.54 in those without del(20q) respectively. Next, we compared PLCG1 expression among MDS subtypes according to the WHO classification in 2008. Median values of the relative PLCG1 expression level in WHO subtypes, RCUD, RCMD, RARS, RAEB‐1, and RAEB‐2 were 1.50, 1.55, 1.29, 1.13, and 1.12, respectively; no significant difference was observed across WHO subtypes. However, patients with high bone marrow blast percentage (5% or more) (RAEB‐1 and RAEB‐2) showed a significantly lower PLCG1 expression level than those with low bone marrow blast percentage (less than 5%) (5q‐, RCUD, RCMD, and RARS) (median value, 0.89 vs 1.60, *P* = .0078; Figure 1C). There was a significant difference in PLCG1 expression among the four IPSS risk groups; the median values of the relative PLCG1 expression level in the low, intermediate‐1, intermediate‐2, and high‐risk groups were 1.55, 1.64, 1.26, and 0.73 respectively (*P* = .0078; Figure 1D).

### 3.3 Reduced PLCG1 expression is associated with lower survival in MDS patients

**Figure 2:**
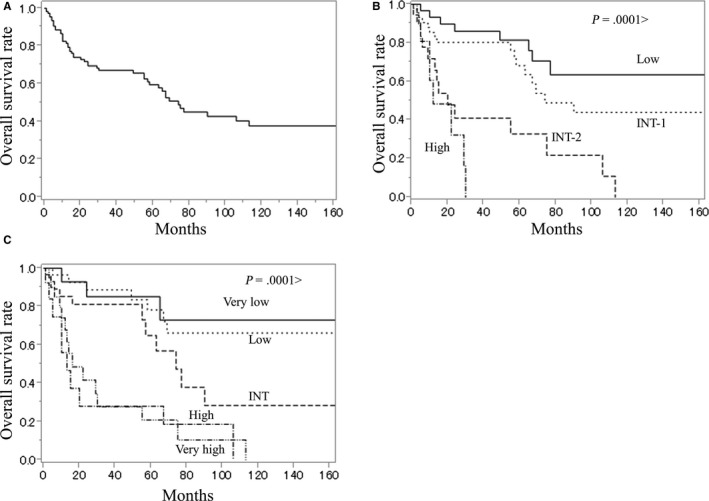
Kaplan‐Meier curves for overall survival in (A) 116 patients, in (B) patients divided into the international prognostic scoring system risk groups, and in (C) patients divided into age‐adjusted revised IPSS risk groups

**Figure 3:**
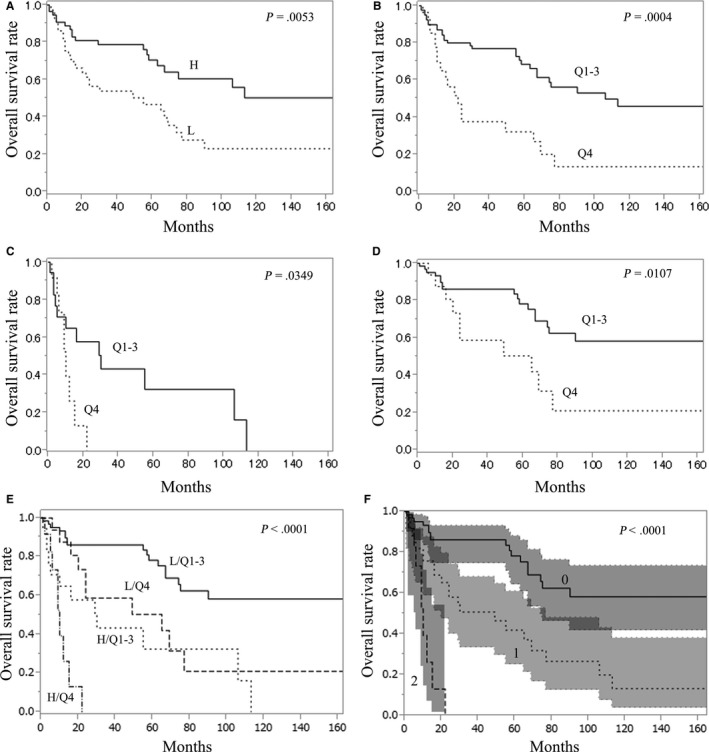
Impact of PLCG1 expression level on overall survival. A, The lower (less than median) PLCG1 expression group (L) had significantly lower overall survival (OS) compared to that of the high (median or higher) PLCG1 expression group (H) (log‐rank test, *P* = .0053). B, The lowest quartile (Q4) group for PLCG1 expression had lower OS compared to that of the other (Q1‐Q3) group (log‐rank test, *P* = .0004). C, Among patients with a high blast percentage (5% or more) (n = 32), patients in the Q4 group had significantly lower OS than those in the Q1‐Q3 group (log‐rank test, *P* = .0349). D, Among patients with a low blast percentage (less than 5%), patients in the Q4 group had significantly lower OS than those in the Q1‐Q3 group (log‐rank test, *P* = .0107). E, Patients were divided into four subgroups according to bone marrow blast percentage, high (5% or more) (H) or low (less than 5%) (L), and PLCG1 expression level, lowest quartile (Q4) or other (Q1‐3). A significant difference in OS was observed among the four groups (log‐rank test, *P* < .0001). F, Patients were stratified into three groups (0, 1, 2) according to the number of risk factors: high bone marrow blast percentage (5% or more) and low PLCG1 expression (the lowest quartile, Q4). A significant difference in OS was observed (log rank test, *P* < .0001). Shaded areas indicate 95% confidence intervals.

To evaluate the prognostic significance of PLCG1 expression at the time of diagnosis, the impact of reduced PLCG1 expression on OS was investigated. The estimated median OS time of the whole cohort (n = 116) was 74 months (Figure 2A; Table 2). By IPSS or age‐adjusted revised IPSS (IPSS‐R) scores, OS was well‐predicted (Figure 2B,C; Table 2). To investigate the impact of PLCG1 expression on OS, 116 patients were divided into two or four subgroups according to the PLCG1 expression level. Kaplan‐Meier plots revealed that patients with lower (less than median) PLCG1 expression had significantly lower OS compared to those with higher (median or higher) PLCG1 expression (log rank test, *P* = .0053) (Figure 3A; Table 3). Among the quartiles divided according to PLCG1 expression, patients in the lowest quartile (Q4) group for PLCG1 expression had the worst OS compared to that of patients in the other groups (Q1‐Q3 groups) (log‐rank test, *P* = .0004) (Figure 3B; Table 3).

**Table 2:** Estimated overall survival rates among patients classified according to IPSS and IPSS‐R

	1‐year	2‐year	5‐year	8‐year
Whole cohort (n = 116)	81.4%	69.5%	59.5%	42.8%
IPSS risk groups				
Low (n = 31)	93.3%	86.0%	81.4%	63.6%
Intermediate‐1 (n = 48)	85.7%	78.0%	66.3%	42.4%
Intermediate‐2 (n = 21)	73.5%	49.0%	33.6%	22.4%
High (n = 13)	48.5%	16.2%	0%	0%
IPSS‐R risk groups				
Very low (n = 16)	92.9%	85.1%	85.1%	73.0%
Low (n = 29)	96.4%	88.7%	78.3%	66.2%
Intermediate (n = 32)	85.2%	81.2%	64.9%	28.4%
High (n = 14)	55.9%	30.0%	30.0%	18.7%
Very high (n = 22)	67.8%	41.6%	20.8%	10.4%

Abbreviations: IPSS, international prognostic scoring system; IPSS‐R, revised international prognostic scoring system.

**Table 3:** Estimated overall survival rates among patients classified according to PLCG1 expression

	1‐year	2‐year	5‐year	8‐year
Whole cohort (n = 116)				
Lower expression (less than median) group (n = 58)	75.4%	56.4%	46.7%	23.0%
Higher expression (median or higher) group (n = 58)	88.8%	80.9%	70.5%	60.5%
The lowest quartile for expression (Q4) (n = 29)	65.1%	37.6%	32.2%	13.4%
Remaining quartiles for expression (Q1‐Q3) (n = 87)	87.1%	80.0%	68.5%	53.1%
Patients with high blast percentage in bone marrow (5% or more) (n = 33)				
The lowest quartile for expression (Q4) (n = 14)	26.2%	0%	0%	0%
Remaining quartiles for expression (Q1‐Q3) (n = 19)	64.9%	57.7%	32.5%	32.5%
Patients with low blast percentage in bone marrow (less than 5%) (n = 83)				
The lowest quartile for expression (Q4) (n = 16)	87.5%	58.7%	50.4%	20.1%
Remaining quartiles for expression (Q1‐Q3) (n = 67)	93.3%	86.2%	78.2%	58.3%

A Cox proportional hazards model revealed that reduced PLCG1 expression (the lowest quartile) was associated with poorer OS by univariate analysis as well as IPSS and IPSS‐R (Table 4). In addition, multivariate analysis showed an association between reduced PLCG1 expression and lower OS. In univariate analyses based on the Cox proportional hazards model, bone marrow blast percentage was also associated with OS. Therefore, we analyzed the impact of reduced PLCG1 expression on OS in each subgroup according to bone marrow blast percentage: high blast percentage (5% or more) or low blast percentage (less than 5%). In patients with high blast percentage (5% or more) (n = 33), the Q4 group had a significantly lower OS than the Q1‐Q3 group (log‐rank test, *P* = .0349) with a median OS time of 10 and 30 months, respectively (Figure 3C; Table 3). The Kaplan‐Meier plots also indicated that the Q4 group had a significantly lower OS than the Q1‐Q3 group in patients with low blast percentage (less than 5%) (log‐rank test, *P* = .0107); the estimated median OS time in the Q4 group was 65 months, and that in the Q1‐Q3 group was not reached (Figure 3D; Table 3). Next, we investigated the combined effect of bone marrow blast percentage and PLCG1 expression on OS. Patients were divided into four subgroups according to blast percentage in the bone marrow, high (5% or more) (H) or low (less than 5%) (L), and PLCG1 expression level, lowest (Q4) or other (Q1‐3) quartiles. Kaplan‐Meier plots indicated a significant difference in OS among the four subgroups: H/Q1‐3 (n = 19), H/Q4 (n = 14), L/Q4 (n = 16), L/Q1‐3 (n = 67) (log‐rank test *P* < .0001); the L/Q1‐3 group had the highest survival, and the H/Q4 group had the lowest survival (Figure 3E; Table 5). Survival curves of the H/Q1‐3 and L/Q4 groups were comparable and intermediate between those of L/Q1‐3 and H/Q4. These results suggest that the lowest quartile of PLCG1 expression (Q4) and high bone marrow blast percentage (5% or more) were risk factors for OS; patients were divided according to number of risk factors and compared for OS. Kaplan‐Meier plots revealed a significant difference in OS among the three groups (log‐rank test, *P* < .0001) (Figure 3F; Table 5).

**Table 4:** Impact of PLCG1 expression and other clinical factors on the overall survival in MDS patients^a^

	Univariate analyses		Multivariate analyses	
	HR (95% CI)	*P*‐value	HR (95% CI)	*P*‐value
PLCG1 expression (the lowest quartile vs other quartiles)	2.78 (1.52‐4.96)	.0012	2.58 (1.35‐4.84)	.0049
Sex (male vs female)	1.15 (0.64‐2.14)	.642		
Age at diagnosis (70 or older vs less than 70)	1.91 (0.99‐3.09)	.053	1.53 (0.84‐2.86)	.17
Bone marrow blast percentage (5% or more vs less than 5%)	3.87 (2.16‐7.02)	<.0001	1.66 (0.73‐3.97)	.23
IPSS risk categories (High, INT‐2 vs INT‐1, low)	4.01 (2.19‐7.27)	<.0001		
IPSS‐R risk categories (Very high and High vs INT, Low, and Very low)	4.77 (2.64‐8.69)	<.0001	3.29 (1.40‐7.30)	.0067

Abbreviations: CI, confidence interval; HR, hazard ratio; INT, intermediate; IPSS, international prognostic scoring system; IPSS‐R, revised IPSS.

a A total of 113 patients in whom risk scores according to IPSS‐R were assessed were analyzed by the COX proportional hazards model.

**Table 5:** Estimated overall survival rates among patients classified according to blast percentage in bone marrow and PLCG1 expression level

	1‐year	2‐year	5‐year	8‐year
Bone marrow blast percentage/PLCG1 expression level^a^				
H/Q1‐3 (n = 19)	64.9%	57.2%	32.5%	32.5%
H/Q4 (n = 14)	26.2%	0%	0%	0%
L/Q1‐3 (n = 67)	93.3%	86.2%	78.2%	58.3%
L/Q4 (n = 16)	87.5%	58.7%	50.4%	21.0%
Number of risk factors^b^				
0 (n = 67)	93.3%	86.2%	78.2%	58.3%
1 (n = 35)	75.8%	58.1%	41.9%	26.6%
2 (n = 14)	26.2%	0%	0%	0%

a Patients were divided four subgroups according to blast percentage in the bone marrow, high (5% or more) (H) or low (less than 5%) (L), and PLCG1 expression level, the lowest (Q4) or other (Q1‐3) quartiles.

b Patients were divided into three subgroups according to number of risk factors we defined: high bone marrow blast percentage (5% or more), and the lowest quartile of PLCG1 expression (Q4).

## 4 DISCUSSION

A significant reduction in PLCG1 expression was observed in MDS patients compared to that of control subjects. Previously, we found that the *PLCG1* gene is located within the CDR of del(20q) observed in MDS.^6^ Haploinsufficiency because of del(20q) may reduce PLCG1 expression, but there were no significant differences in expression levels of PLCG1 between MDS patients with del(20q) and those without del(20q), which suggests that reduced PLCG1 expression is not specific in MDS patients with del(20q), but a common molecular event in MDS. The molecular mechanism of reduced PLCG1 expression in MDS patients without del(20q) remains unclear. The most plausible explanation is that reduced PLCG1 expression in MDS without del(20q) is because of epigenetic dysregulation of the *PLCG1* gene. Recent advances in understanding the molecular genetics of MDS revealed the importance of epigenetic dysregulation of genes involved in cell growth, death, and differentiation.^15^, ^16^ The clinical efficacy of hypomethylating agents also suggests that epigenetic dysregulation is important in the molecular pathogenesis of MDS. In addition, cumulative evidences indicate importance of splicing factor gene mutations, which are most frequent mutations in MDS patients, in molecular pathogenesis of MDS.^17^, ^18^ Mutations of splicing factor genes may affect global gene expression and splicing, resulting in alteration of expression level and/or splicing in diverse genes, which may include the *PLCG1* gene, involved in molecular pathogenesis of MDS.

Reduced PLCG1 expression is associated with a lower OS in MDS patients. Univariate and multivariate analyses based on the Cox proportional hazards model revealed that reduced PLCG1 expression at the time of diagnosis is an independent prognostic biomarker for MDS. PLCG1 expression was reduced in MDS with a high blast percentage (5% or more) compared to those with a low blast percentage (less than 5%), which suggests that reduced PLCG1 has a role in the progression of MDS. Reduced PLCG1 expression may simply reflect bone marrow blast percentage, and the prognostic significance of reduced PLCG1 expression at the time of diagnosis is associated with advanced MDS. We found that reduced PLCG1 expression in subgroups divided by bone marrow blast percentage, high blast percentage (5% or more) and low blast percentage (less than 5%), was significant for prognosis. By using both factors, bone marrow percentage (5% or more) and reduced PLCG1 expression (lowest quartile), patients were well‐stratified for OS, and OS was predicted by these two factors at the time of diagnosis. It is important to determine a standardized cut‐off value for the clinical application of PLCG1 expression as a prognostic biomarker, and further investigation of PLCG1 expression in MDS patients is needed.

The biological significance of reduced PLCG1 expression in MDS has not been determined. The *PLCG1* gene encodes phospholipase C γ1, which has multiple roles in physiological and pathological intracellular pathways through nuclear inositide signaling and is involved in the development of human cancers including hematological malignancies.^19^, ^20^, ^21^ Reduced PLCG1 expression may result in the dysregulation of phospholipase C γ1‐mediated cellular process. Phospholipase C‐mediated nuclear inositide signaling is important in the pathogenesis of MDS.^13^ Deletion of the *PLCB1* gene, which encodes another phospholipase C member, phospholipase C β1, is associated with leukemic transformation in MDS.^14^ Reduced PLCB1 expression is observed in MDS and associated with clinical outcomes.^22^ Although the functional difference between PLCG1 and PLCB1 is not clear, reduced expression of phospholipase C members may be involved in MDS molecular pathogenesis. PLCG1 is involved in hematopoiesis and possibly in pathological hematopoiesis including MDS. Animal models in which PLCG1 is disrupted suggest that PLCG1 is involved in hematopoiesis.^11^, ^12^ In addition, molecular biological analyses suggest that PLCG1 is involved in erythropoiesis. Erythropoiesis is a strictly regulated cellular process, and multiple pathways are involved. Janus kinase 2 (JAK2) plays a central role in erythropoietin receptor (EPOR) signaling, which is an important intracellular signaling pathway for erythrocyte proliferation and differentiation.^23^, ^24^ PLCG1 functions as a downstream molecule of JAK2 without depending on STAT5, which is involved in the EPOR‐JAK2 pathway in normal and pathological erythropoiesis.^25^ STAT5 is a transcription factor and induces the expression of genes involved in cell survival, cell proliferation, and cell cycle progression including CyclinD1 and BCL‐xL.^24^, ^26^ Plcg1‐deficient mice have severe defects in erythroid differentiation, but STAT5‐mediated processes, including cell survival and proliferation, are not affected.^25^ Therefore, reduced expression of PLCG1 may result in impaired erythroid differentiation and maturation in MDS. Recombinant human erythropoietin is used to treat anemia in low‐risk MDS patients. Interestingly, PLCG1‐mediated inositide signaling pathways are activated by recombinant EPO treatment in low‐risk MDS patients, which suggests that PLCG1 is involved in erythropoiesis in MDS.^27^ In addition to the enzymatic activity of phospholipase C, PLCG1 physically interacts with other molecules through its SH2 domains and modulates their functions.^8^, ^28^ In growth hormone receptor signaling, PLCG1 regulates JAK2‐STAT5 pathways by forming complexes with JAK2 and protein tyrosine phosphatase‐1B (PTP‐1B).^29^ In this complex, PTP‐1B negatively regulates JAK2 function by dephosphorylating JAK2 tyrosine residues, which downregulates STAT5. Although it is not clear whether similar molecular mechanisms are involved in EPOR‐mediated intracellular pathways, PTP‐1B dysfunction may cause dysregulation in proliferation and survival. PLCG1 may function not only as a downstream molecule but also a regulator of JAK2 in the EPOR‐JAK2 pathway, which suggests a complex role for PLCG1 in normal and pathological erythropoiesis. Interestingly, the *PTPN1* gene, which encodes PTP‐1B, is located within the CDR of del(20q).^6^ It is possible that the *PTPN1* gene is also a target gene that is disrupted by chromosome deletion in the molecular pathogenesis of MDS.

We found that reduced *PLCG1* expression was associated with worse clinical outcomes, and the level of *PLCG1* expression at the time of MDS diagnosis was a useful prognostic marker. To understand the biological significance of reduced PLCG1 expression in MDS molecular pathogenesis, functional analysis is needed.
